# Psychological Factors, Including Alexithymia, in the Prediction of Cardiovascular Risk in HIV Infected Patients: Results of a Cohort Study

**DOI:** 10.1371/journal.pone.0054555

**Published:** 2013-01-22

**Authors:** Giustino Parruti, Francesco Vadini, Federica Sozio, Elena Mazzott, Tamara Ursini, Ennio Polill, Paola Di Stefano, Monica Tontodonati, Maria C. Verrocchio, Mario Fulcheri, Giulio Calella, Francesca Santilli, Lamberto Manzoli

**Affiliations:** 1 Unit of Infectious Diseases, Pescara General Hospital, Pescara, Italy; 2 Clinical Psychology Division, University of Chieti-Pescara, Italy; 3 Department of Medicine and Aging, University of Chieti-Pescara, Italy; 4 Section of Hygiene, Epidemiology, Pharmacology and Legal Medicine, University of Chieti-Pescara, Italy; University Medical Center Utrecht, The Netherlands

## Abstract

**Background:**

Psychological factors are known predictors of cardiovascular disease in many clinical settings, but data are lacking for HIV infection. We carried out a prospective cohort study to evaluate potential psychological predictors of preclinical and clinical vascular disease in HIV patients.

**Methodology/Principal Findings:**

HIV patients were consecutively enrolled. Demographics, viral and immune parameters and traditional cardiovascular predictors were considered; Intima-Media Thickness (c-IMT, continuous measure) and Carotid Plaques (CPs, focal thickening ≥1.5 mm) were investigated by B-mode ultrasonography; depressive symptoms by the Beck Depression Inventory (BDI-II), Type D personality (Distressed Personality or Type D) by the DS14, alexithymia by the Toronto Alexithymia Scale (TAS-20). Vascular outcomes included transient ischemic attacks or stroke, acute coronary syndrome, myocardial or other organ infarction. We enrolled 232 HIV subjects, 73.9% males, aged 44.5±9.9 y, 38.2% with AIDS diagnosis, 18.3% untreated. Mean Nadir CD4 T-cell counts were 237.5±186.2/mmc. Of them, 224 (96.5%) attended IMT measurements; 201 (86.6%) attended both IMT assessment and psychological profiling. Mean follow-up was 782±308 days. Fifty-nine patients (29.4%) had CPs at baseline. Nineteen patients (9.5%) had ≥1 vascular event; 12 (6.0%) died due to such events (n = 4) or any cause. At baseline cross-sectional multivariate analysis, increasing age, total cholesterol, current smoking and Alexithymia score≥50 were significantly associated with both increased cIMT (linear regression) and CPs (logistic regression). At follow-up analysis, log-rank tests and Cox’s regression revealed that only older age (p = 0.001), current smoking (p = 0.019) and alexithymia score≥50 (p = 0.013) were independently associated with vascular events.

**Conclusions/Significance:**

In HIV-infected subjects, the Alexithymic trait emerges as a strong predictor of increased IMT, presence of CPs and vascular events. Such results are preliminary and require confirmation from studies with larger sample size and longer follow-up.

## Introduction

HIV-infected individuals are known to be at higher risk of cardiovascular disease (CVD) than the general population [1], [2]. HIV infection per se has been associated with imbalance of inflammatory cytokines, endothelial dysfunction [3], [4], hypercoagulability states [5], [6] and vascular damage [7], [8]. The sharp decline of HIV associated morbidity and mortality in the era of Highly Active Antiretroviral Therapy (HAART) was somewhat counterbalanced by the emergence of a number of metabolic derangements, including insulin resistance, frank abnormalities of glucose metabolism, dyslipidemias and changes in body fat distribution [9], [10]. As compared with uninfected controls, increased intima-media thickness of carotid arteries (c-IMT) and faster progression of atheromasic lesions have been more frequently observed in HIV-infected patients [11]–[14], and an increased risk of myocardial infarction (MI) and other vascular events during continual HAART has been conclusively demonstrated [15], [16].

So far, preventive strategies have focused on modifiable traditional risk factors for CVD [17]. Although the link between psychological factors, such as depression, Type D personality (Distressed Personality or Type D) and alexithymia, and atherosclerosis or vascular events has been repeatedly evaluated in patients with heart diseases and in the general population, it has been poorly examined in the HIV-infected population [18]–[21]. Type D consists of two traits: negative affectivity (NA), the tendency of an individual to experience negative emotions, and social inhibition (SI), the tendency to feel discomfort in social interactions, to exhibit a lack of social poise, and to avoid confrontation. Alexithymia is a “multi-trait” personality construct characterized by a deficiency in the cognitive processing of emotions, namely, difficulties in identifying and communicating emotions, and externally-oriented thinking. These characteristics reflect a disruption in the conscious experience of emotions. Psychological factors may be related to atherosclerosis and other vascular events through their association with behavioral risk factors, such as smoking, physical activity, diet and abdominal obesity [22], [23]. They may also directly affect biological processes by multiple pathways, such as inflammation [24], cardiovascular reactivity [25], endothelial injury [26], [27], platelet activation [28], [29] and autonomic dysfunction [30], [31], whereby psychological factors may play both a primary and a secondary pathogenetic role [32].

For this reason, the contextual evaluation of both psychological and traditional cardiovascular risk factors may represent a useful widening of the investigation of vascular risk also in the HIV infected population. In 2007, we therefore designed a single center cohort study, to evaluate the role of psychological factors, including alexithymia, Type D and depressive symptoms, in parallel with several traditional predictors of increased c-IMT, Carotid Plaque(s) (CPs) and ensuing vascular events in HIV-infected patients. Here we report the results of baseline evaluation and the first two-year follow-up, indicating that the alexithymic trait, characterized by an impaired cognitive processing of emotional stimuli, may be a relevant, previously unraveled, predictor of CVD and vascular events in this setting.

## Methods

### Study Design

The study included a cross-sectional analysis of the baseline data to evaluate the predictors of increased c-IMT and CPs, and a prospective cohort analysis to explore the determinants of cardiovascular events during a 5-year follow-up.

### Demographic, Clinical and Viro-immunological Characterization of the Sample

Inclusion criteria were: treated or untreated confirmed chronic HIV infection; age ≥18 y; any CD4 T-cell counts and HIV viremia; absence of acute opportunistic infections, malignancy or pregnancy at the time of enrollment and subsequent study procedures; sufficient knowledge of the Italian language to undergo psychological profiling; willingness and ability to provide written informed consent. All consecutive HIV infected patients aged 18 y or more, attending the Outpatient Clinics of the Infectious Disease Unit of Pescara General Hospital, Italy, were offered participation at their first access. The study psychologist was on duty in most of the days when HIV outpatients were cared for and he was the only staff member authorized to perform study presentation and psychological tests. Therefore, patients were offered participation in the study (psychological profiling and c-IMT measurements) only when the psychologist was present. On purpose accesses for participants were kept to a minimum, study procedures being performed at one of the next scheduled visits whenever possible. Laboratory measurements were obtained at accesses for either study procedure.

At the baseline visit, each patient agreeing to participate underwent an in-depth assessment, including a structured interview covering socio-demographic characteristics, HIV disease history, presence of other comorbid conditions, health-related behaviors, smoking status, cardiovascular risk factors, including diabetes [33], medication exposure, and family or personal history of vascular events (acute myocardial infarction (AMI), Transient Ischemic Attack (TIA) or stroke). The 10-year cardiovascular risk was assessed using the Framingham risk score (FRS) model, recommended by NCEP (National Cholesterol Education Program), as accessed at http://hp2010.nhlbihin.net/atpiii/calculator.asp [34].

AIDS-defining events, HCV and/or HBV coinfection(s), other relevant comorbid condition(s), concomitant medications, duration of HIV infection, duration of HIV treatment, Nadir and current CD4 T-cell Lymphocyte counts, HIV RNA levels at diagnosis and at enrollment, use of Tenofovir (TDF) and third antiretroviral drug (LPV/r, ATV/r, other boosted PIs, EFV, NVP) were also recorded.

Additional information is provided as online supplemental material (Methods S1).

### Definition of the Main Outcomes

The main outcomes of our investigation were: (1) c-IMT assessed as a continuous variable; (2) presence of CPs at baseline; (3) vascular events during follow-up (every 3 months), including TIA or stroke, acute coronary syndrome (unstable Angina, non-ST Elevation MI and STEMI) and myocardial or other organ infarctions. All ultrasonographic scannings of carotid arteries of the short and long axis were performed by the same certified operator [35], [36]. Vascular events were actively searched in the cohort. They were positively assessed at each follow-up visit by the assisting physicians and recorded in the study dataset when appropriate, after review of clinical, laboratory and imaging data supporting diagnoses of each vascular event. Records of all patients dying during follow-up were also reviewed before inclusion in the dataset. Finally, in order to avoid unnecessary data censoring, as of March 20^th^, 2012, all patients not completing their last follow-up visit (n = 38) were interviewed by phone, to assess the possibility of late vascular events. Additional information is provided in the online supplemental material (Methods S1).

### Definitions and Measures of the Psychological Constructs

The three psychological constructs were assessed contextually by the same operator within a single interview. Depression symptoms were investigated through the Beck Depression Inventory II (BDI-II) [37]. Type D is a relatively new construct characterized by 2 global personality traits: negative affectivity (NA) and social inhibition (SI) [38], [39]. This personality trait was assessed with the Italian version of the 14-item DS14, consisting of 2 7-item subscales for NA and SI [39]. Alexithymia is a disorder of the regulatory mechanisms of emotional and cognitive processing, characterized by the following impairments: difficulty in differentiating feelings and distinguishing them from bodily sensations and emotional arousal (DIF), difficulty in describing feelings to others (DDF), and an externally oriented way of thinking (EOT) [40], [41]. We used the Italian version of the TAS-20 (Toronto Alexithymia Scale), universally employed for the alexithymia construct worldwide [41]. Additional information is provided in the online supplemental material (Methods S1).

### Statistical Analysis - Cross-sectional Analysis of Baseline Data

Both multivariable linear and logistic regression analyses were used to evaluate potential independent predictors of increased c-IMT of carotid arteries and the presence of CPs. The dependent variable was IM thickening as measured by IMT as a continuous variable in the linear models and the presence of plaque(s) as a dichotomic variable in the logistic models. In linear regression analysis, the dependent variable was the inverse of IMT, which was transformed because of its skewed distribution (Shapiro–Wilk). We defined the regression models including a priori potential confounders (age, gender, BMI, smoking, hypertension, diabetes, one among total cholesterol and LDL-cholesterol, physical activity, alcohol/drug abuse, educational level, presence of psychiatric disorders and infection duration), because of their known association with IMT, and including other eventually significant variables, which were selected using a stepwise forward process (including in model fitting all variables that were significant at a 0.10 level in univariate analyses). Each covariate was tested in its original form or transformed if needed. In addition, each variable included was tested for multicollinearity, for potential interaction and/or quadratic/cubic terms. We found only total and LDL cholesterol to be collinear and chose to include total cholesterol. For continuous covariates for which a defined threshold has been indicated in the literature (i.e. TAS-20 score ≥50), we tested the inclusion of both the continuous and categorical form, and selected the one that was included in the model with highest pseudo or adjusted R-squared values. The procedures to assess the validity of the logistic and linear models are described in detail in the online supplemental material (Methods S1).

For both linear and logistic models, all variables were tested for inclusion and all variables that were not included into the final models were not significant. Also, we tried to limit as much as possible the number of covariates included in the final model to avoid overfitting, and among the non-significant variables we left inside the final model those that were associated with the highest increase in model pseudo or adjusted R-squared, or that changed the significant coefficients by more than 10%.

The results of the logistic analysis are presented as odds ratio (OR) and 95% confidence limits whereas the results of the linear regression analysis are presented as beta-coefficients, their standard errors and standardized coefficients in order to quantify the relative contribution of each covariate to the prediction of IMT.

### Statistical Analysis – Survival Analysis of Follow-up Data

Cox proportional hazards analysis was used to calculate the adjusted relative hazards of a vascular event by each variable. Stochastic level of entry into the model was set at 0.10, and interaction terms were explored for all variables in the final model. A minimum events-to-variable ratio of 10 was maintained in multivariate modeling to avoid overfitting, and Schoenfeld’s test was performed to check the validity of proportional hazards assumption [42]. We forced to entry all variables that were significantly (or border line) associated with increased c-IMT and CPs in the linear and logistic regression models, respectively. As for the latter models, all variables were tested for inclusion and all variables that were not included into the final Cox model were not significant. Kaplan-Meier survival analysis was used to examine the association between a TAS-20 score ≥50 and other significant variables and vascular event. The validity of constant incidence ratios over the follow-up was checked using Nelson-Aalen cumulative hazard estimates [43].

In all multivariate analyses, less than 5% of the participants had missing data, thus no missing data imputation technique was adopted. Statistical significance was defined as a two-sided *p*-value<0.05 for all analyses, which were performed using STATA 10.1 (Stata Corp., College Station, TX, U.S.A., 2007).

### Ethics Statement

The study protocol was set up and filed for approval by the local Ethical Committee (Comitato etico per la Sperimentazione Clinica dei Farmaci ASL di Pescara, http://www.comitatoeticopescara.it/statuto.html) and Health District Authorities early in 2007. The final approval (authorization n° 943) was granted on August 28^th^, 2008. Patients were enrolled and followed from April 2007 through March, 2012. Informed consent was signed by patients at enrollment, both to undergo study investigations and for anonymised publishing of data.

## Results

### Study Sample

**Figure 1:**
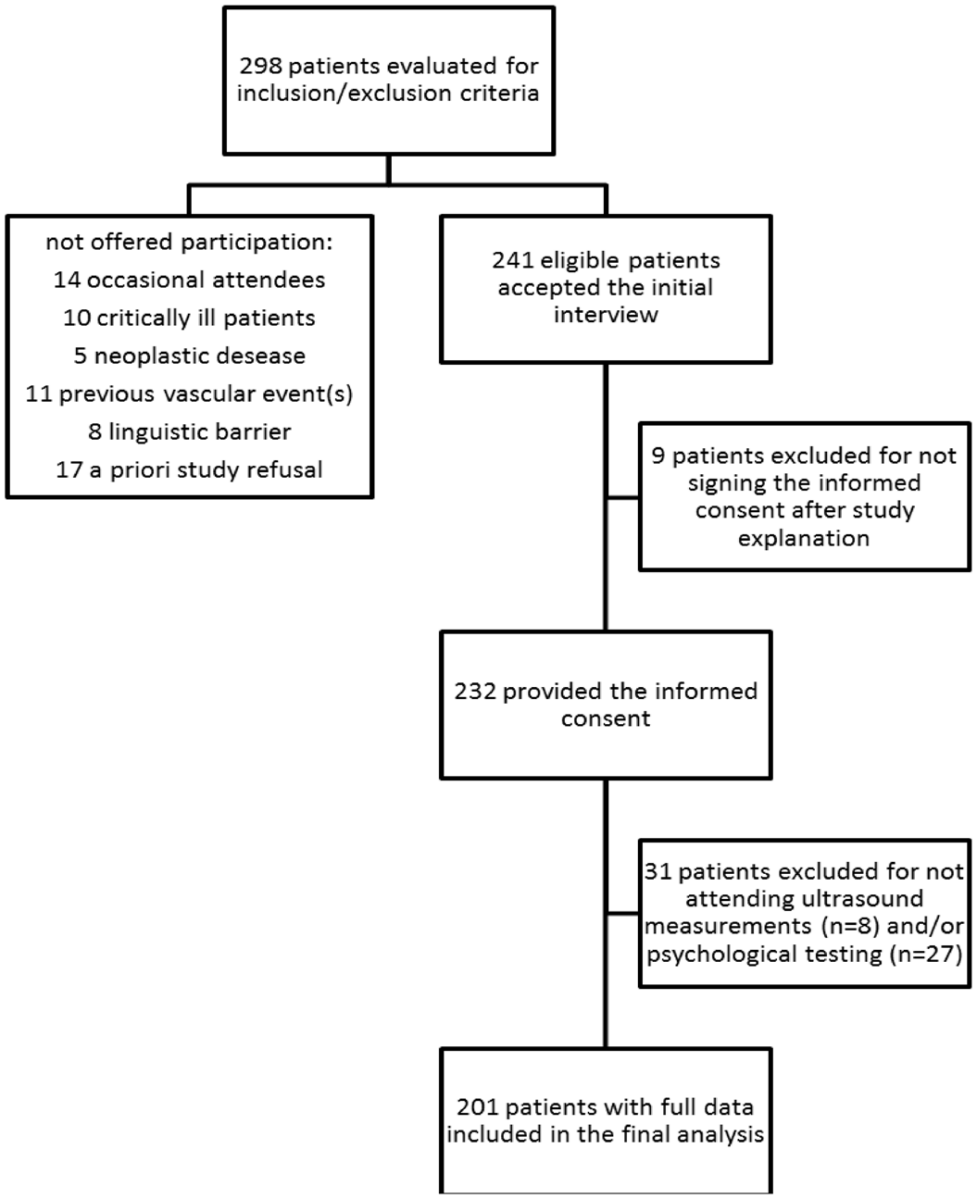
Eligibility Flow-Chart.

The initial cohort screened for eligibility to participate in the study consisted of 298 patients (Figure 1). Fourteen HIV-infected patients visiting occasionally our Center were not offered participation. Fifteen more patients were AIDS presenters with severe clinical deterioration (n = 10) or chronic HIV patients with hematological or neoplastic diseases (n = 5); similarly these patients were not offered participation. Eight patients were excluded due to linguistic barriers; 11 patients were not included as they reported previous cardiovascular events; finally, a priori refusal of the initial interview was expressed by 17 patients.

During the enrollment period, 241 eligible patients accepted the initial interview and 232 also provided the informed consent. Of them, 224 (96.6%) attended IMT measurements; 205 (88.4%) psychological testing; 201 (86.6%) both procedures and could thus be included in the multivariate analyses. The 31 patients with missing values in one or more of the main outcomes were unsuccessfully rescheduled for baseline visits more than once. To exclude relevant selection biases, we compared the intention-to-treat and per-protocol samples. In the total (n = 232) and final (n = 201) samples, respectively, males were 74.1% and 76.0%; the mean age was 44.5±9.9 y and 45.1±10.1 y; drug abusers were 25.1% and 25.0%; heterosexuals 43.5% and 41.7%; homosexuals 28.9% and 31.3% and transfusions 2.5% and 2.0% (all p>0.05). None of the other parameters, including AIDS diagnosis, proportion of treatment naïve patients and mean Nadir CD4 T-cell counts, were significantly different across samples.

The clinical, behavioral and socio-demographic characteristics of the final sample (n = 201) are described in Table 1.

**Table 1:** Clinical, behavioral and socio-demographic characteristics of the final sample (n = 201).

Category	
Age, mean (SD)	45.1 (10.1)
Gender	
Male, n (%)	153 (76.1)
Duration of HIV infection, yr mean (SD)	5.2 (4.4)
HCV co-infection, n (%)	54 (26.9)
AIDS diagnosis, n (%)	76 (37.8)
HAART-treated, n (%)	160 (80)
Duration of HAART, yr, mean (SD)	5.1 (4.3)
Other drug(s), ≥1, n (%)	89 (59.8)
Nadir CD4 T-cell counts, mean (SD)	234 (181)
CD4 T-cell counts at baseline, mean (SD)	499 (283)
HIV viremia, undetectable at baseline, n (%)	120 (67.0)
Body Mass Index, mean (SD)	24.20 (3.82)
Hypertension, any level, treated or not, n (%)	53 (26.4)
Lipodystrophy, any type, n (%)	91 (45.3)
Framingham Risk Score, n (%)	
0–10	149 (74.1)
10–20	41 (20.4)
>20	11 (5.5)
Total Cholesterol, mmol/L, mean (SD)	5.0 (1.54)
Triglycerides, mmol/L, mean (SD)	1.94 (1.40)
HDL Cholesterol, mmol/L, mean (SD)	1.07 (0.34)
Fasting plasma glucose, mmol/L	5.15 (1.43)
Diabetes, n(%)	36 (17.9)
Intima Media Thickness, mm, mean (SD)	0.57 (0.22)
Presence of carotid plaque (≥1.5 mm focal thickening),n (%)	59 (29.4)
Alexithymia TAS-20 score, n (%)	
20–49	116 (57.7)
50–60	48 (23.8)
≥61	37 (18.5)
Alexithymia TAS-20≥50, n (%)	85 (42.3)
Alexithymia TAS-20≥61, n (%)	37 (18.5)
Type D Personality, n (%)	68 (34.2)
Beck Depression Inventory, ≥17, n (%)	56 (27.9)
Current Smoking, n (%)	113 (56.2)
Regular Sport practice, n (%)	70 (35)
Employed, n (%)	138 (69)
Married, n (%)	84 (41.8)
Psychiatric disorder, n (%)	27 (13.4)
Active drug abuse, n (%)	21 (10.5)
Educational level, n (%)	
High	48 (23.9)
Medium	77 (38.3)
Low	76 (37.8)
Drug use	
Tenofovir, n (%)	136 (67.7)
NNRTI, n (%)	68 (33.8)
Nevirapine, n (%)	26 (12.9)
Efavirenz, n (%)	44 (21.9)
PIs, n (%)	91 (45.3)
Lopinavir, n (%)	27 (13.4)
Atazanavir, n (%)	37 (18.4)
Other PIs, n (%)	31 (15.6)

### Predictors of c-IMT (Cross-sectional Analysis)

At baseline, among the 201 patients in the final sample, mean c-IMT was 0.57±0.22 mm. Fifty-nine patients (29.4%) were found to have at least one vascular carotid lesion meeting the primary (≥1.5 mm) definition of CP [36].

As shown in Table 2, at univariate analyses increased c-IMT was significantly associated with older age, duration of HIV infection, AIDS diagnosis, treatment with HAART and its duration, treatment with other drugs, hypertension, lipodystrophy, higher Framingham risk score, total cholesterol, fasting plasma glucose, TAS-20 score ≥50. As shown in Table 3, the presence of CPs showed similar predictors: older age, duration of HIV infection, AIDS diagnosis, HAART duration, therapy with other drug(s), hypertension, lipodystrophy and higher Framingham scores, smoking, higher triglycerides and fasting plasma glucose, diabetes, alexithymia (at both cut-off values, TAS-20≥50 and ≥61), nevirapine as 3^rd^ drug. Type D and a depression BDI score ≥17 were not significantly associated with CPs, whereas an higher educational level provided significant protection. A border-line significance was observed for HAART therapy, higher cholesterol levels, lower Nadir CD4 T-cell counts, presence of psychiatric disorders.

**Table 2:** Intima Media Thickness (IMT) in the sample (n = 201), according to selected variables.

	n.	Mean IMT	SD	P (Rho)*
Age class				<0.001 (0.35)
Age <50 y	140	0.53	0.19	
Age 50–64 y	53	0.67	0.25	
Age ≥65 y	8	0.76	0.29	
Gender				0.7
Male	153	0.56	0.03	
Female	48	0.58	0.02	
Duration of HIV infection				0.01
<10 y	64	0.52	0.17	
≥10 y	136	0.60	0.24	
HCV co-infection				0.06
Yes	54	0.53	0.14	
No	147	0.59	0.24	
AIDS diagnosis				0.025
Yes	76	0.62	0.27	
No	125	0.55	0.18	
HAART				0.008
Treated	160	0.60	0.23	
Untreated	40	0.49	0.13	
Duration of HAART				0.020 (0.16)
Naïve	37	0.49	0.13	
<1 y	11	0.55	0.18	
1–7 y	87	0.59	0.23	
>7 y	66	0.60	0.25	
Other drug(s)				0.030
≥1	89	0.62	0.25	
None	60	0.53	0.16	
Nadir CD4 T-cell count, cell/mmc				0.4 (−0.05)
<100	56	0.60	0.27	
100–250	59	0.56	0.14	
>250	82	0.58	0.24	
CD4 T-cell count at baseline, cell/mmc				0.6 (0.03)
<250	36	0.55	0.17	
250–500	80	0.57	0.22	
>500	83	0.59	0.24	
HIV viremia				0.2
Undetectable Detectable	120 59	0.60 0.55	0.24 0.19	
Body Mass Index, kg/m^2^				0.3 (0.07)
<20	12	0.58	0.23	
20–25	115	0.57	0.24	
26–30	60	0.59	0.19	
>30	14	0.56	0.17	
Hypertension				0.007
Any level, treated or not	53	0.64	0.27	
Normotensive	148	0.55	0.20	
Lipodystrophy				0.001
Any type	91	0.63	0.28	
No	110	0.53	0.15	
Framingham Risk Score				<0.001 (0.28)
0–9	144	0.53	0.16	
10–20	46	0.65	0.28	
>20	11	0.78	0.43	
Total Cholesterol, mmol/L				0.003 (0.21)
<5.17	122	0.53	0.15	
5.17–6.18	47	0.61	0.29	
≥6.2	32	0.67	0.29	
Triglycerides, mmol/L				0.17
≤1.69	107	0.55	0.22	
>1.69	92	0.60	0.22	
HDL Cholesterol, mmol/L				0.1 (0.12)
<1.03	103	0.54	0.15	
1.03–1.52	71	0.63	0.29	
≥1.55	21	0.57	0.18	
Fasting plasma glucose, mmol/L				0.03
≤6.11	171	0.56	0.20	
>6.11	26	0.67	0.35	
Diabetes				0.15
Yes	36	0.62	0.29	
No	165	0.56	0.20	
Alexithymia TAS-20 score				0.046 (0.15)
20–50	121	0.54	0.16	
51–60	42	0.62	0.26	
≥61	38	0.64	0.31	
Alexithymia TAS-20≥50				<0.001
Yes	85	0.65	0.30	
No	116	0.53	0.13	
Alexithymia TAS-20≥61				0.06
Yes	37	0.64	0.31	
No	164	0.56	0.20	
Type D Personality (DS-14)				0.12
Yes	68	0.62	0.24	
No	131	0.55	0.021	
Depression (BDI-II)				0.22
≥17	56	0.54	0.15	
<17	145	0.59	0.24	
Smoking				0.5
Current smoking	113	0.58	0.26	
Former or never smoking	88	0.56	0.16	
Sport practice				0.15
Yes	70	0.54	0.16	
No	130	0.59	0.25	
Occupation				0.09
Employed	138	0.56	0.18	
Unemployed	62	0.61	0.29	
Familial status				0.9
Married	84	0.58	0.19	
Not married	117	0.57	0.24	
Psychiatric disorder				0.7
Yes	27	0.56	0.18	
No	174	0.58	0.23	
Active drug abuse				0.4
Yes	21	0.54	0.13	
No	180	0.58	0.23	
Educational level				0.5 (0.04)
High	76	0.57	0.23	
Medium	77	0.57	0.23	
Low	48	0.58	0.20	
Drug use				
Tenofovir, Yes	136	0.57	0.24	0.8
Tenofovir, No	65	0.58	0.19	
NNRTI, Yes	68	0.60	0.23	0.3
NNRTI, No	133	0.56	0.22	
Nevirapine, Yes	26	0.63	0.21	0.13
Nevirapine, No	175	0.56	0.22	
Efavirenz, Yes	44	0.56	0.22	0.7
Efavirenz, No	157	0.58	0.23	
PIs, Yes	91	0.59	0.24	0.3
PIs, No	110	0.56	0.21	
Lopinavir, Yes	27	0.56	0.16	0.7
Lopinavir, No	174	0.58	0.23	
Atazanavir, Yes	37	0.54	0.16	0.3
Atazanavir, No	164	0.58	0.23	
Other PIs, Yes	31	0.63	0.32	0.17
Other PIs, No	168	0.57	0.20	

* p-values of the comparison between groups (categorical variables: chi-squared test; continuous variables: Kruskal-Wallis test). For continuous variables only, the Spearman Rho of the correlation between IMT and the variable in its continuous form has been reported in brackets.

**Table 3:** Characteristics of the sample according to the presence of atherosclerotic plaque (IMT_max_ ≥1.5 mm).

	Plaque	No Plaque	p*
	(n = 59)	(n = 142)	
Mean age in years mean(SD)	51.9 (9.4)	41.3 (9.0)	<0.001
Male gender, %	81.4	74.0	0.3
Duration of HIV infection, y, mean (SD)	15.7 (7.3)	12.2 (7.2)	0.002
HCV co-infection, %	28.8	26.1	0.7
AIDS diagnosis, %	50.9	32.4	0.014
Treated with HAART, %	88.1	76.6	0.06
Duration of HAART, y (SD)	6.5 (4.4)	4.6 (4.2)	0.003
Other drug(s), %**	79.6	51.4	0.001
Nadir CD4 T-cell count, cell/mmc (SD)	197 (180)	250 (180)	0.06
CD4 T-cell count at baseline, cell/mmc (SD)	498 (273)	500 (288)	0.9
HIV viremia, detectable, %	31.6	33.6	0.8
Body mass index, Kg/m^2^, mean (SD)	24.53	24.10	0.4
Hypertension, %	45.8	18.3	<0.001
Lipodystrophy, %	66.1	36.6	<0.001
Framingham Risk Score mean (SD)	12.4 (8.3)	4.5 (5.2)	<0.001
Total cholesterol, mmol/L mean (SD)	5.33 (1.53)	4.88 (1.53)	0.06
HDL-c, mmol/L mean (SD)	1.1 (0.35)	1.10 (0.33)	0.11
Triglycerides, mmol/L mean (SD)	2.41 (1.96)	1.74 (1.03)	0.002
Fasting plasma glucose, mmol/L mean (SD)	5.59 (1.34)	4.97 (1.43)	0.005
Diagnosis of Diabetes, %	30.5	12.7	0.003
Alexithymia			
Mean TAS-20 score mean (SD)	52.5 (14.3)	45.2 (13.6)	<0.001
TAS-20≥50, %	62.7	33.8	<0.001
TAS-20≥61, %	33.9	12.6	<0.001
Depression BDI score >17, %	33.9	25.4	0.2
Type D Personality, %	37.3	32.9	0.6
Gainful employment, %	61.0	72.3	0.1
Smoker, %	67.8	51.4	0.03
Sport practice, %	30.5	36.9	0.4
Married, %	44.1	40.9	0.7
Active drug abuse, %	11.9	9.9	0.7
Psychiatric disorders, %	20.3	10.6	0.06
Educational level, %			0.04
Low	50.8	32.3	
Medium	32.2	40.9	
High	20.8	26.7	
Therapy			
Tenofovir, %	64.4	69.0	0.5
NNRTI, %	40.7	31.0	0.2
Nevirapine, %	22.0	9.2	0.01
Efavirenz, %	18.6	23.2	0.5
PIs, %	44.1	45.8	0.8
Lopinavir, %	13.6	13.4	0.9
Atazanavir, %	11.9	21.1	0.12
Other PIs, %	22.0	12.9	0.10

* Kruskal-Wallis test for continuous variables; chi-squared test for categorical ones.

** 59 missing values (21 among those with plaque; 38 among other participants).

At multivariate analyses, only increasing age, total cholesterol, current smoking and a TAS-20≥50 remained significantly associated with both cIMT (Table 4) and CPs (Table 5). Our findings on CPs were similar in the logistic models using three different thresholds for carotid plaque (≥1.5 mm; ≥1.2 mm; ≥1.0 mm), with the exception of current smoking, that was not significant with the least conservative threshold for plaque (1.0 mm). In particular, subjects with a TAS-20≥50 consistently showed an OR >2.5 of having a CP (p<0.01) with all thresholds (Table 5). Even at the higher cut-off score for alexithymia (TAS-20≥61), the association between alexithymia and CPs remained significant (p<0.02 whatever the c-IMT_max_ threshold for plaque; data not shown). Notably, no other factor among those identified in univariate analyses was independently associated with either cIMT or CPs.

**Table 4:** Results from the multivariable regression model evaluating the association between the inverse of carotid Intima Media Thickening (c-IMT) and various explanatory factors.

	Regression coefficient	(95% CI)	p	Standardized coefficient
Age, 5-year increase	−0.086	(−0.129; −0.044)	<0.001	−0.309
Male gender	0.030	(−0.153; 0.213)	0.8	0.022
Body Mass Index, one unit increase	−0.006	(−0.030; 0.017)	0.6	−0.040
Infection duration, years	−0.008	(−0.019; 0.004)	0.2	−0.099
Hypertension	−0.008	(−0.211; 0.196)	0.9	−0.005
Current smoking	−0.076	(−0.233; 0.082)	0.3	−0.065
Diabetes mellitus	0.049	(−0.168; 0.266)	0.7	0.032
Total cholesterol, 10 mg/dL increase	−0.022	(−0.035; −0.009)	0.001	−0.225
Alexithymia TAS−20 score ≥50	−0.164	(−0.322; −0.007)	0.032	−0.141

CI = Confidence Interval. The final sample included 196 patients.

**Table 5:** Results from the logistic regression models predicting the presence of plaques (using diverse cutoffs for focal thickening) with Alexithymia TAS-20 score cut-off ≥50.

	IMT_max_≥1.5 mm			IMT_max_≥1.2 mm			IMT_max_≥1.0 mm		
Variables	OR	(95% CI)*	p	OR	(95% CI)*	p	OR	(95% CI)*	p
Age, 5-year increase	1.72	(1.36–2.19)	<0.001	2.25	(1.73–2.93)	<0.001	2.03	(1.58–2.62)	<0.001
Male gender	2.24	(0.74–6.75)	0.15	2.12	(0.72–6.26)	0.17	1.93	(0.71–5.26)	0.2
Body Mass Index, 1 unit increase	0.99	(0.88–1.10)	0.8	1.02	(0.91–1.15)	0.7	0.96	(0.85–1.09)	0.5
Infection duration, 1-year increase	1.04	(0.98–1.10)	0.19	1.05	(0.99–1.12)	0.082	1.05	(0.99–1.11)	0.077
Hypertension	1.93	(0.78–4.82)	0.16	1.73	(0.67–4.46)	0.3	2.59	(0.91–7.35)	0.074
Current smoking	2.74	(1.16–6.44)	0.021	2.48	(1.04–5.92)	0.041	1.55	(0.66–3.63)	0.3
Diabetes mellitus	1.10	(0.37–3.23)	0.9	1.15	(0.37–3.55)	0.8	1.02	(0.32–3.23)	0.9
Total cholesterol, 10 mg/dL increase	1.10	(1.05–1.16)	<0.001	1.12	(1.07–1.18)	<0.001	1.12	(1.06–1.18)	<0.001
Alexithymia TAS-20 score ≥50	2.63	(1.17–5.89)	0.019	2.99	(1.31–6.83)	0.009	4.23	(1.86–9.61)	0.001

CI = Confidence Interval. OR = Odds Ratio.

* Based upon robust standard errors. Parameters of the models: (1.0 mm) n. of obs. = 196; Wald chi-squared = 66.4; Hosmer–Lemeshow goodness of fit p = 0.27; area under the receiving operator curve (ROC) = 0.87; (1.2 mm) n. of obs. = 196; Wald chi-squared = 61.9; Hosmer–Lemeshow goodness of fit p = 0.68; area under the receiving operator curve (ROC) = 0.87; (1.5 mm) n. of obs. = 196; Wald chi-squared = 44.1; Hosmer–Lemeshow goodness of fit p = 0.23; area under the receiving operator curve (ROC) = 0.84.

### Predictors of Vascular Events (Follow-up Survival Analysis)

The mean follow-up of the 201 patients undergoing both c-IMT and psychological evaluations was 782±308 days (for a total of 430.5 person-years). The overall incidence of vascular events was 4.41 (95% CI: 2.66–6.89) per 100 person-years. Ninety-nine patients (9.5%) had a vascular event during the follow-up: 6 AMI, 6 TIA, 5 strokes, and 2 other organ infarctions (one intestinal infarction and 1 renal infarction). Deaths from any cause were 12; of these, 4 (33.3%) were caused by cardiovascular events.

The distributions of all considered variables were compared in patients with or without cardiovascular events (Table 6). At univariate analyses, age, c-IMT, presence of plaque, Framingham score and alexithymia score (both continuous or dichotomic) were significantly associated with vascular events. Also, a border-line significance was observed for smoking. All other factors were not significantly associated with vascular events. Notably, the incidence of vascular events was 8.45 (95% CI: 4.73–13.9) per 100 person-years in patients with TAS-20≥50; 1.58 (95% CI: 0.43–4.05) per 100 person-years in patients with TAS<20.

**Table 6:** Characteristics of the final sample according to the presence of vascular events.

	Vascular	No	p*
	Events	Events	
	(n = 19)	(n = 182)	
Mean age, y, mean (SD)	54.1 (8.5)	44.2 (9.8)	<0.001
Male gender, %	9.8	8.3	0.8
Duration of HIV infection, y, mean (SD)	14.6 (7.3)	13.1 (7.4)	0.4
HCV co-infection, %	5.6	10.9	0.3
AIDS diagnosis, %	13.2	7.2	0.16
Treated with HAART, %	9.4	7.5	0.7
Duration of HAART, y, mean (SD)	5.7 (4.0)	5.1 (4.4)	0.6
Other drug(s), % **	11.2	5.0	0.2
Nadir CD4 T-cell count, cell/mmc mean (SD)	281 (244)	229 (173)	0.23
CD4 T-cell count at baseline, cell/mmc, mean (SD)	557 (317)	493 (280)	0.4
HIV viremia, detectable (%)	10.2	10.0	1.0
Body Mass Index mean (SD)	24.0 (3.2)	24.2 (3.9)	0.4
Lipodystrophy, %	13.2	6.4	0.1
Hypertension, %	13.2	8.1	0.3
Diagnosis of Diabetes, %	11.1	9.1	0.7
Fasting plasma glucose, mmol/L mean (SD)	5.3 (0.72)	5.1 (1.5)	0.8
Total cholesterol, mmol/L, mean (SD)	5.3 (1.6)	5.0 (1.5)	0.4
HDL-c, mmol/L, mean (SD)	1.1 (0.47)	1.1 (0.33)	0.7
Triglycerides, mmol/L, mean (SD)	1.94 (1.3)	1.93 (1.4)	1.0
Framingham Risk Score mean (SD)	13.8 (9.3)	6.1 (6.6)	<0.001
c-IMT, mm mean (SD)	0.76 (0.33)	0.55 (0.20)	<0.001
Presence of plaque (≥1.5 mm), %	25.4	2.8	<0.001
Alexithymia			
Mean TAS-20 score mean (SD)	53.5 (14.0)	46.7 (14.0)	0.05
TAS ≥50, %	17.7	3.5	0.001
Depression BDI score>17, %	14.3	7.6	0.14
Type D Personality, %	11.8	7.6	0.30
Current smoking, %	12.4	5.7	0.1
Sport practice, %	5.7	11.5	0.2
Gainful employment, %	8.7	11.3	0.6
Married, %	8.3	10.3	0.6
Active drug abuse, %	4.8	10.0	0.4
Psychiatric disorders, %	11.1	9.2	0.8
Educational level, %			
Low	52.6	36.3	0.16
Medium	31.6	39.0	0.5
High	15.8	24.7	0.4
Therapy			
Tenofovir, %	8.1	12.3	0.4
NNRTI, %	14.7	6.8	0.07
Nevirapine, %	19.2	8.0	0.07
Efavirenz, %	6.8	10.2	0.5
PIs, %	6.6	11.8	0.2
Lopinavir, %	7.4	9.8	0.7
Atazanavir, %	10.8	9.2	0.8
Other PIs, %	9.7	8.9	0.9

* Kruskal-Wallis test for continuous variables; chi-squared test for categorical ones.

**Figure 2:**
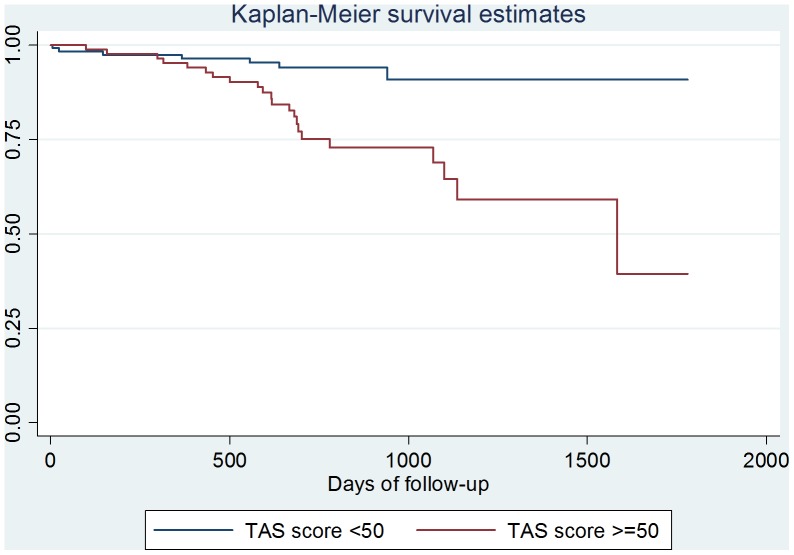
Kaplan-Meier estimates of time to vascular events by alexithymia (TAS-20 score ≥50 versus TAS-20 score <50).

In the Cox proportional hazards model, age (Hazard ratio, HR, 1.55, 95%CI: 1.19–2.02, p = 0.001), current smoking (3.87, 1.25–12.0, p = 0.019) and TAS-20≥50 (5.58, 1.44–21.6, p = 0.013) were the only factors significantly associated with vascular events (Table 7). The relationship between vascular events and alexithymia is also apparent from the shape variation of the Kaplan-Meier estimates of time to event (Figure 2).

**Table 7:** Results from the Cox proportional hazards regression analyses predicting time to vascular events.

	Hazard Ratio	(95% CI)	p
Age, 5-year increase	1.55	(1.19–2.02)	0.001
Infection duration, 1-year increase	0.98	(0.91–1.05)	0.5
Hypertension	0.44	(0.13–1.50)	0.2
Current smoking	3.87	(1.25–12.0)	0.019
Total cholesterol, 10 mg/dL increase	1.06	(0.98–1.14)	0.13
Alexithymia TAS-20 score ≥50 vs <50	5.58	(1.44–21.6)	0.013

## Discussion

Depression, Type D and alexithymia were found to be strongly related to cardiovascular risk in several populations [44]–[48], but HIV patients were not yet considered. This cohort study investigated for the first time the potential role of such psychological traits, in addition to traditional cardiovascular risk factors, in predicting CVD in the HIV population.

Despite an association between CVD and depression was repeatedly documented, and several studies showed a role of immune parameters in such a relationship, making its investigation particularly interesting in the setting of HIV infection [49], [50], we observed a possible association between higher depression scores and the presence of CPs in univariate models, but failed to demonstrate an independent association when contextually evaluating the other psychological constructs.

Some prospective studies suggested a potential association of Type D with hypercholesterolemia, hypertension, metabolic syndrome and increased mortality rates in patients with established CVD [51]–[56]. As for depression, the association among Type D personality and investigated outcomes was not confirmed in our cohort.

In contrast, we found that higher alexithymia scores were associated with higher c-IMT, presence of CPs and vascular events, both in univariate analyses and in all final multivariate models. Indeed, several studies investigated the relationships between alexithymia, cardiovascular risk factors, CVD and cardiovascular mortality in the general population [44], [45], [57], [58]. In particular, two large population-based studies linked the alexithymic trait with a previous diagnosis of coronary heart disease and subclinical atherosclerosis and, more recently, with increased cardiovascular mortality [44], [45]. The preliminary, mechanistic explanation of such findings may be provided by the fact that subjects with the alexithymic trait were shown to suffer amplified and prolonged neurovegetative reactions to stress stimuli, using models of skin-conductance response [59], [60]. Such investigations suggested that the alexithymic trait may disturb the autonomic system and the pituitary-adrenal axis, leading to increased neuroendocrine insult of the vasculature [44], [60]. In fact, in the HIV setting, the association between the alexithymic trait and CVD risk is in line with the predictions of the cognitive-emotional interaction model [61]–[65], according to which the high prevalence of alexithymia in HIV may reflect the effects of the virus on sub-cortical areas of the brain, involved in cognitive emotional regulation and the relative autonomic responses mediating vascular damage [66]–[68].

An alternative explanation for the observed association between alexithymia and CVD would be that alexithymic patients might have an intrinsic higher risk of CVD simply because they more frequently present traditional cardiovascular risk factors. For instance, if subjects with alexithymia were more commonly diabetics, hypertensive, obese and smokers, their higher risk of CVD would not be caused by the psychological trait, but rather by such well-known risk factors (a typical example of confounding). Also, in this scenario, the co-inclusion of such factors and alexithymia in multivariate analysis might have covered the statistical significance of single risk factors due to a certain degree of multicollinearity (which may also explain why we did not observe a significant association between CVD and other well-known CVD risk factors). However, in our sample alexithymic patients were not significantly more hypertensive (p = 0.6), smokers (p = 0.2) or obese (p = 0.2). They were older (48.3 y vs 42.8 y, p<0.001), and more frequently diabetics (27.1% vs 11.2%, p = 0.004). Nevertheless, when analyses were stratified by age-class and diabetes, alexithymia remained significantly and strongly associated with the presence of plaques as well as with vascular events both in patients <50 y and in non diabetics (see Table S1).

Our data would therefore suggest a true association between the alexithymic trait and CVD risk in HIV patients, in agreement with the above mentioned cognitive-emotional interaction model [61]–[68]. Alexithymia may thus represent a novel and unexpected determinant of accelerated atherothrombosis in the setting of HIV infection.

Importantly, in our sample 42.3% of the patients had a TAS-20 score ≥50; a prevalence of the alexithymic trait including borderline cases that is nearly three times higher than in the general population [69], [70]. Such proportion rose to 70.4% in patients aged ≥55 (p = 0.002, data not shown). Thus, besides the relatively small sample size and short follow-up, our data can only provide preliminary findings, to be interpreted with caution, because the high prevalence of the alexithymic trait may partly reflect a secondary effect of HIV-mediated vascular damage on such brain areas [66], [71], [72]. On the other side, if the observed association will be confirmed, the high prevalence of alexithymia in our sample may explain the high incidence of vascular events that we observed in a relatively short follow-up: approximately 10% of patients suffered a vascular event (lethal in one third of cases), a remarkable and unexpected rate at the time of cohort setting.

It is worth noting, in any case, that this is the first evaluation of the prevalence of alexithymia in an unselected sample of HIV patients. Notably, the alexithymic trait was not associated with either current (p = 0.8) or Nadir CD4 T-cell counts (p = 0.9), duration of HIV infection (p = 0.8), AIDS diagnosis (p = 0.5) or baseline HIV viremia (p = 0.4, data not shown), suggesting either a relation with behaviors at risk of getting HIV infection, such as drug addiction [73], or with the early phases of HIV infection [68]. Furthermore, data collected on a fraction (n = 70) of patients in our sample, who were retested for psychological traits after a mean of 18 months, revealed that alexithymic scores were only minimally modified (data not shown), indicating a remarkable stability of such a psychological phenotype over time, in line with other reports in the general population [74].

The major strengths of our study are the accurate consecutive enrolment of the study sample, the contextual evaluation of both the psychological traits and c-IMT, each operated by a single certified investigator, and the active follow-up for vascular events, that allowed limiting data censoring to a minimum in the final Cox’s multivariate models. In addition to the limitations mentioned above, shortcomings of the study are the single experimental site and the inclusion of 95% of Caucasian subjects.

In conclusion, HIV alexithymic patients may be at a significantly and persistently increased risk of both preclinical and clinically overt vascular damage. Such a result is preliminary and require confirmation from studies with larger sample size and longer follow-up. In fact, further research is strongly warranted: should our findings be confirmed, they may pave the way to an array of additional interventions to control cardiovascular disorders in the HIV population.

## Supporting Information

Table S1 **Association between presence of plaque or vascular events and alexithymia (TAS-20 score ≥50), stratified by age class and diabetes.**
(DOC)

Methods S1 **Supplementary methods.**
(DOC)
