# Correction: Psychological Factors, Including Alexithymia, in the Prediction of Cardiovascular Risk in HIV Infected Patients: Results of a Cohort Study

**DOI:** 10.1371/annotation/ee9c345b-884e-412c-a455-f73df01c6df5

**Published:** 2014-01-22

**Authors:** Giustino Parruti, Francesco Vadini, Federica Sozio, Elena Mazzotta, Tamara Ursini, Ennio Polilli, Paola Di Stefano, Monica Tontodonati, Maria C. Verrocchio, Mario Fulcheri, Giulio Calella, Francesca Santilli, Lamberto Manzoli

The names of the fourth and sixth authors were spelled incorrectly. The correct names are: Elena Mazzotta and Ennio Polilli. The correct citation is: 

Parruti G, Vadini F, Sozio F, Mazzotta E, Ursini T, et al. (2013) Psychological Factors, Including Alexithymia, in the Prediction of Cardiovascular Risk in HIV Infected Patients: Results of a Cohort Study. PLoS ONE 8(1): e54555. doi:10.1371/journal.pone.0054555.

