# Supplementary material for: Psychological Factors, Including Alexithymia, in the Prediction of Cardiovascular Risk in HIV Infected Patients: Results of a Cohort Study
Source: PLoS One. 2013 Jan 22;8(1):e54555. doi: 10.1371/journal.pone.0054555 (PMC3551818; doi:10.1371/journal.pone.0054555)
Supplement: Table S1 — Association between presence of plaque or vascular events and alexithymia (TAS-20 score ≥50), stratified by age class and diabetes. (DOC) [file pone.0054555.s001.doc]

**Table S1. Association between presence of plaque or vascular events and alexithymia (TAS-20 score ≥50), stratified by age class and diabetes**

| *Stratification* |  | Plaque, % | p | Plaque, % | Vascular Events, % | p | Vascular Events, % |
| --- | --- | --- | --- | --- | --- | --- | --- |
| *category* |  | TAS-20 ≥50 |  | TAS-20<50 | TAS-20 ≥50 |  | TAS-20<50 |
| Age <50y | (n=140) | 29.4 | 0.01 | 12.4 | 11.8 | 0.005 | 1.1 |
| Age 50-64y | (n=53) | 59.3 | 0.13 | 38.5 | 25.9 | 0.08 | 7.7 |
| Age ≥ 65y | (n=8) | 85.7 | 0.7 | 100 | 28.6 | 0.2 | 100 |
| Diabetes | (n=36) | 56.5 | 0.3 | 38.5 | 13.0 | 0.6 | 7.7 |
| No Diabetes | (n=165) | 38.61 | 0.001 | 12.40 | 19.4 | <0.001 | 2.9 |
