# Supplementary material for: Psychological Factors, Including Alexithymia, in the Prediction of Cardiovascular Risk in HIV Infected Patients: Results of a Cohort Study
Source: PLoS One. 2013 Jan 22;8(1):e54555. doi: 10.1371/journal.pone.0054555 (PMC3551818; doi:10.1371/journal.pone.0054555)
Supplement: Methods S1 — Supplementary methods. (DOC) [file pone.0054555.s002.doc]

**Methods S1**

*Demographic, clinical and viro-immunological characterization of the sample*

At the baseline visit, each patient agreeing to participate underwent an in-depth assessment, including a structured interview covering socio demographic characteristics, HIV disease history, presence of other comorbid conditions, health-related behaviors, medication exposure, and family history. Patients were characterized for: age, sex, BMI, blood pressure, use of antihypertensive drugs (hypertension was defined as a confirmed systolic BP of ≥140 mmHg and/or diastolic BP of ≥90 mmHg, or intake of one or more antihypertensive medications), familial history of cardiovascular events (early acute myocardial infarction (AMI), transient ischemic attack (TIA) or stroke, smoking status, fasting plasma glucose and presence of diabetes (diagnosed according to current guidelines [1]), total cholesterol and triglyceride levels, AIDS-defining events, HCV (defined as the presence of HCV antibodies and at least a positive determination of HCV viremia, obtained during follow-up in the absence of treatment) and/or HBV coinfection(s), other relevant comorbid condition(s), concomitant medications, duration of HIV infection, duration of HIV treatment, Nadir and current CD4 T-cell lymphocyte counts, HIV RNA levels at diagnosis and at enrollment, use of Tenofovir (TDF) and third antiretroviral drug (LPV/r, ATV/r, other boosted PIs, EFV, NVP). Diagnoses of hypertension were confirmed and followed with the expert contribution of a consultant cardiologist in all cases. The 10-year cardiovascular risk was assessed using the Framingham risk score (FRS) model, recommended by NCEP (National Cholesterol Education Program), as accessed at http://hp2010.nhlbihin.net/atpiii/calculator.asp [2].

*Definition of the main outcomes*

The main outcomes of our investigation were: (1) c-IMT assessed as a continuous variable; (2) presence of CPs at baseline; (3) vascular events during follow-up, including TIA or stroke, acute coronary syndrome (unstable angina, non-ST Elevation MI and STEMI), myocardial and other infarctions. All ultrasonographic scannings of carotid arteries of the short and long axis were performed by the same certified operator, with the patient lying in a supine position and its head extra-rotated from the opposite side. IMT of both common carotid arteries (c-IMT) was measured with a high resolution B-mode ultrasound imager (ESAOTE; Siemens Corporation, Paris, France) on the carotid arterial far wall with a linear array transducer of frequency at 7.5 MHz and a pulsed Doppler; internal and external branches of the carotid artery were explored whenever imaged. Examination included both measurement of c-IMT in regions free of plaque and assessment of presence of CPs on the explored segments. c-IMT was calculated as the mean of at least 3 measurements of the double-line pattern wall, considered at least 1 cm from bifurcation, because of its validated association with cardiovascular risk [3], and registered as a continuous measure. CPs were defined as the presence of a focal thickening ≥ 1.5 mm regardless of the generalized wall thickening of the explored trait(s) and whatever the echoic pattern (hypoechoic, isoechoic or hyperechoic), or as the presence of a focal wall thickening at least 0.5 mm or 50% greater than that of the surrounding vessel wall [3,4]. However, in line with previous reports on HIV patients and in the general population, CPs were also scored either in the presence of a focal thickening ≥ 1.2 mm [5,6] or even in the presence of a focal thickening ≥ 1.0 mm [7]. In other terms, CPs were scored according to all possible thresholds in use for both HIV patients and the general population. Vascular events were actively searched in the cohort. They were positively assessed at each follow-up visit (on average every 3 months) by the assisting physicians and recorded in the study dataset when appropriate, after review of clinical, laboratory and imaging data supporting diagnoses of each vascular event. Records of all patients dying during follow-up were also reviewed before inclusion in the dataset. Finally, in order to avoid unnecessary data censoring, as of March 20^th^, 2012, all patients not completing their last follow-up visit (n=38) were interviewed by phone, to assess the possibility of late vascular events.

*Definitions and measures of the psychological constructs*

The three psychological constructs were assessed contextually by the same operator within a single interview. Depression symptoms were investigated through the Beck Depression Inventory II (BDI-II). The BDI-II is a self-report inventory for adolescents and adults that assesses the presence and severity of depressive symptoms experienced during the past 2 weeks [8]. The BDI-II includes 21 items, each consisting of 4 statements reflecting varying degrees of symptom severity [8,9]. BDI scores were registered as a continuum, and dichotomized at ≥17, higher scores being recognized as indicative of moderate to severe symptoms of depression. Type D personality is a relatively new construct characterized by 2 global personality traits: negative affectivity (NA) and social inhibition (SI) [10,11]. Persons with this type of personality are inclined to negative emotions which are further associated with the tendency to inhibit these emotions by avoiding social contacts [12]. This personality trait was assessed with the Italian version of the 14-item DS14, consisting of 2 7-item subscales for NA and SI [12,13], which was extensively used to assess type-D patterns in relationship with cardiovascular events in cardiac patients and in the general population [14]. A score of ≥ 9 on both NA and SI subscales is considered suggestive that an individual has Type D [13]. Alexithymia is a disorder of the regulatory mechanisms of emotional and cognitive processing, characterized by the following impairments: difficulty in differentiating feelings and distinguishing them from bodily sensations and emotional arousal (DIF), difficulty in describing feelings to others (DDF), and an externally oriented way of thinking (EOT) [15,16]. The prevalence of individuals with clinically relevant alexithymia was estimated as 10% to 13% in Western populations [17-19]. We used the Italian version of the TAS-20 (Toronto Alexithymia Scale), universally used for the Alexithymia construct worldwide [20-24]. This questionnaire consists of 20 items answered on a 5-point scale, targeting the 3 specific dimensions of DIF, DDF and EOT. Alexithymia scores are normally distributed in the general population. For our analyses, we first considered individual alexithymia scores as a continuous measure; we also dichotomized our population in accordance with the 2 different cut-offs extensively validated for moderate or severe alexithymia (TAS score ≥ 50) and severe alexithymia (TAS score ≥ 61) [21].

*Statistical analysis – Additional information on the cross-sectional analysis of baseline data*

In logistic regression analysis, the outlier analysis was based upon the calculation of Pearson and standardized residuals, the change in Pearson chi-square and deviance chi-square, Dbeta influence statistic and leverage (hat diagonal matrix) [25]. The validity of the final linear regression model was assessed as follows. The assumption of constant error variance was checked graphically, plotting Pearson residuals vs. fitted values, and formally, using the Cook-Weisberg test for heteroskedasticity. High leverage observations were identified by computing Pearson, standardized and studentized residuals, Cook's D influence, Welsch distance and the hat diagonal matrix. We found 13-16 influential observations in the logistic model and 7-8 high-leverage observations in the linear model. In both cases, we repeated the analysis excluding these observations, with no substantial changes. Given that the number of outliers were above 5% of the total number of participants, the final logistic model was conservatively based upon robust standard errors.

**References**

1. American Diabetes Association. (2011) Standards of medical care in diabetes--2011. Diabetes Care 34: S11-S61.

2. National Cholesterol Education Program (NCEP) Expert Panel (ATP III). (2002) Third report of the National Cholesterol Education Program (NCEP) Expert Panel on Detection, Evaluation, and Treatment of High Blood Cholesterol in Adults (Adult Treatment Panel III) final report. Circulation 106: 3143-3421.

3. O’Leary DH, Polak JF. (2002) Intima-media thickness: a tool for atherosclerosis imaging and event prediction. Am J Cardiol 90: 18–21.

4. [Stein JH](http://www.ncbi.nlm.nih.gov/pubmed?term=Stein JH%5BAuthor%5D&cauthor=true&cauthor_uid=18261694), [Korcarz CE](http://www.ncbi.nlm.nih.gov/pubmed?term=Korcarz CE%5BAuthor%5D&cauthor=true&cauthor_uid=18261694), [Hurst RT](http://www.ncbi.nlm.nih.gov/pubmed?term=Hurst RT%5BAuthor%5D&cauthor=true&cauthor_uid=18261694), [Lonn E](http://www.ncbi.nlm.nih.gov/pubmed?term=Lonn E%5BAuthor%5D&cauthor=true&cauthor_uid=18261694), [Kendall CB](http://www.ncbi.nlm.nih.gov/pubmed?term=Kendall CB%5BAuthor%5D&cauthor=true&cauthor_uid=18261694), et al. (2008) Use of carotid ultrasound to identify subclinical vascular disease and evaluate cardiovascular disease risk: a consensus statement from the American Society of Echocardiography Carotid Intima-Media Thickness Task Force. Endorsed by the Society for Vascular Medicine. [J Am Soc Echocardiogr](http://www.ncbi.nlm.nih.gov/pubmed/18261694) 21:93-111.

5. Jericò C, Knobel H, Calvo N, Sorli ML, Guelar A, et al. (2006) Subclinical Carotid Atherosclerosis in HIV-Infected Patients. Role of Combination Antiretroviral Therapy. Stroke;37:812-817.

6. Yerly P, [Rodondi N](http://www.ncbi.nlm.nih.gov/pubmed?term=Rodondi N%5BAuthor%5D&cauthor=true&cauthor_uid=23015308), [Viswanathan B](http://www.ncbi.nlm.nih.gov/pubmed?term=Viswanathan B%5BAuthor%5D&cauthor=true&cauthor_uid=23015308), [Riesen W](http://www.ncbi.nlm.nih.gov/pubmed?term=Riesen W%5BAuthor%5D&cauthor=true&cauthor_uid=23015308), [Vogt P](http://www.ncbi.nlm.nih.gov/pubmed?term=Vogt P%5BAuthor%5D&cauthor=true&cauthor_uid=23015308), et al. (2012) Association between conventional risk factors and different ultrasound-based markers of atherosclerosis at carotid and femoral levels in a middle-aged population. [Int J Cardiovasc Imaging.](http://www.ncbi.nlm.nih.gov/pubmed/23015308) Sep 27 doi: <http://dx.doi.org/10.1007/s10554-012-0124-3>

7. Masiá M, Padilla S, Robledano C, Ramos JM, Gutiérrez F. (2011) Evaluation of endothelial function and subclinical atherosclerosis in association with hepatitis C virus in HIV-infected patients: a cross-sectional study. BMC Infectious Diseases;11:265.

8. Beck AT, Steer RA, Brown GK eds. (1996) Manual for the Beck Depression Inventory-II. San Antonio, TX: Psychological Corporation.

9. American Psychiatric Association eds. (2000) Diagnostic and Statistical Manual of Mental Disorder, 4th Edition, Text Revision. Washington, DC: American Psychiatric Association.

10. Denollet J. (2000) Type D personality. A potential risk factor refined. J Psychosom Res 49: 255-266.

11. De Fruyt F, Denollet J. (2002) Type D personality: A Five Factor Model perspective. Psychol Health 17: 671-683.

12. Denollet, J. (2005) DS14: Standard assessment of negative affectivity, social inhibition, and Type D personality. Psychosom Med 67: 89–97.

13. Kupper N, Denollet J. (2007) Type D personality as a prognostic factor in heart disease: assessment and mediating mechanisms. J Pers Assess 89: 265-276.

14. Grande G, Romppel M, Vesper JM, [Schubmann R](http://www.ncbi.nlm.nih.gov/pubmed?term=Schubmann R%5BAuthor%5D&cauthor=true&cauthor_uid=21862827), [Glaesmer H](http://www.ncbi.nlm.nih.gov/pubmed?term=Glaesmer H%5BAuthor%5D&cauthor=true&cauthor_uid=21862827), et al. (2011) Type D personality and all-cause mortality in cardiac patients--data from a German cohort study. Psychosom Med 73: 548-56.

15. Sifneos PE. (1973) The prevalence of alexithymic characteristics in psychosomatic patients. Psychother Psychosom 22: 255–262.

16. Taylor GJ, Bagby RM, Parker JD. (1991) The alexithymia construct. A potential paradigm for psychosomatic medicine. Psychosomatics 32:153–164.

17. Joukamaa M, Taanila A, Miettunen J, [Karvonen JT](http://www.ncbi.nlm.nih.gov/pubmed?term=Karvonen JT%5BAuthor%5D&cauthor=true&cauthor_uid=17905044), [Koskinen M](http://www.ncbi.nlm.nih.gov/pubmed?term=Koskinen M%5BAuthor%5D&cauthor=true&cauthor_uid=17905044), et al. (2007) Epidemiology of Alexithymia among adolescents. J Psychosom Res 63: 373-376.

18. Salminen JK, Saarijärvi S, Aärelä E, [Aärelä E](http://www.ncbi.nlm.nih.gov/pubmed?term=Aärelä E%5BAuthor%5D&cauthor=true&cauthor_uid=10088984), [Toikka T](http://www.ncbi.nlm.nih.gov/pubmed?term=Toikka T%5BAuthor%5D&cauthor=true&cauthor_uid=10088984), et al. (1999) Prevalence of alexithymia and its association with sociodemographic variables in the general population of Finland. J Psychosom Res 46: 75-82.

19. Honkalampi K, Hintikka J, Tanskanen A, [Lehtonen J](http://www.ncbi.nlm.nih.gov/pubmed?term=Lehtonen J%5BAuthor%5D&cauthor=true&cauthor_uid=10750635), [Viinamäki H](http://www.ncbi.nlm.nih.gov/pubmed?term=Viinamäki H%5BAuthor%5D&cauthor=true&cauthor_uid=10750635). (2000) Depression is strongly associated with alexithymia in the general population. J Psychosom Res 48: 99-104.

20. Bagby RM, Parker JDA, Taylor GJ. (1994) The 20-item Toronto alexithymia scale-1. Item selection and cross-validation of the factor structure. J Psychosom Res 38: 23-32.

21. Bressi C, Taylor G, Parker J, [Bressi S](http://www.ncbi.nlm.nih.gov/pubmed?term=Bressi S%5BAuthor%5D&cauthor=true&cauthor_uid=9032718), [Brambilla V](http://www.ncbi.nlm.nih.gov/pubmed?term=Brambilla V%5BAuthor%5D&cauthor=true&cauthor_uid=9032718), et al. (1996) Cross validation of the factor structure of the 20-item Toronto Alexithymia Scale: an Italian multicenter study. J Psychosom Res 41: 551-559.

22. Megank R, Vanheule S, Desmet M. (2008) Factorial validity and measurement invariance of the 20-item Toronto Alexithymia Scale in clinical and nonclinical samples. Assessment 15: 36-47.

23. Parker J D A, Taylor G J, Bagby RM. (2003) The 20-item Toronto Alexithymia Scale -III. Reliability and factorial validity in a community population. J Psychother Pract Res 55: 269-275.

24. Tolmunen T, Heliste M, Lehto SM. (2011) Stability of alexithymia in the general population: an 11-year follow-up. Compr Psychiatry 52:536–541.

25.Hosmer DW, Lemeshow S eds. (2000) Applied Logistic Regression, 2nd Edition. New York: John Wiley & Sons.
